# Utilization and Expenditures of High-Need, High-Cost Older Adults With Disabilities

**DOI:** 10.1093/geroni/igab046.601

**Published:** 2021-12-17

**Authors:** Sarita Karon, Cleanthe Kordomenos, Molly Knowles, Micah Segelman

**Affiliations:** 1 RTI International, Waltham, Massachusetts, United States; 2 RTI International, Research Triangle Park, North Carolina, United States

## Abstract

The factors that lead people to have high needs for care can vary greatly, with implications for the best approaches to serving their needs. One high need group of interest is older adults with disabilities and multiple comorbidities. There is variation in need within this group. Of particular interest is the subset that is both high need and high cost (HNHC). We present work describing Medicare and Medicaid utilization and expenditures for this high need group and the HNHC subset. Over 7.6 million people were identified as high need; 13.6% of them also were defined as HNHC. Patterns of utilization differed between these groups, with the HNHC group more likely to use inpatient care and nursing home care, but less likely to use community-based long-term services and supports. These findings have implications for the development of care models that might best meet the needs of this population.

